# Supplementary material for: The Construction and Exploration of a Comprehensive MicroRNA Centered Regulatory Network in Foxtail Millet (Setaria italica L.)
Source: Front Plant Sci. 2022 May 6;13:848474. doi: 10.3389/fpls.2022.848474 (PMC9121102; doi:10.3389/fpls.2022.848474)
Supplement: Supplementary file 5 [file Data_Sheet_5.zip › Sit-miRNovel5_Seita.2G294000.1_10966_TPlot.pdf]

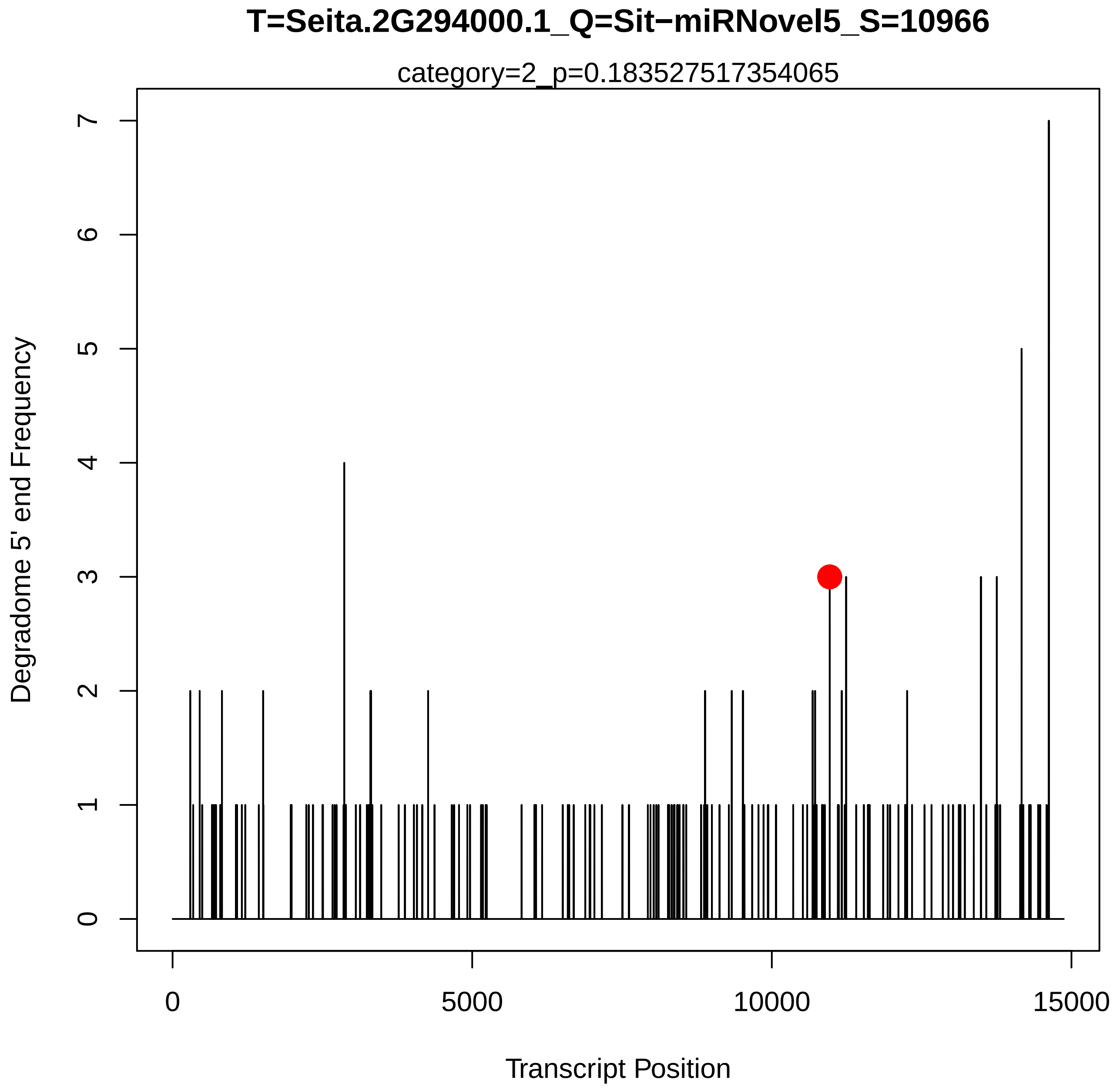
T=Seita.2G294000.1_Q=Sit−miRNovel5_S=10966
category=2_p=0.183527517354065
Degradome 5' end Frequency
0
1
2
3
4
5
6
7
0
5000
10000
15000
Transcript Position
